# Allergen characterization in childhood asthma: a retrospective cohort study in China

**DOI:** 10.3389/fpubh.2025.1692904

**Published:** 2025-11-12

**Authors:** Chong Hu, Qin Dong, Yanzi Zhang, Juan Wang, Dan Wu, Xin Lv

**Affiliations:** 1 Clinical Laboratory, Children's Hospital Affiliated to Shandong University, Jinan, China; 2 Clinical Laboratory, Jinan Children's Hospital, Jinan, China

**Keywords:** childhood asthma, inhalant allergens, food allergens, specific IgE, sensitization pattern

## Abstract

**Background:**

The increasing cases of asthma in children around the world necessitate the profiling of regional specific allergens to manage it precisely. This research study identifies the sensitization patterns of Chinese children with asthma and the necessity of its undertaking for the prevention of this chronic respiratory illness that is prevalent in children.

**Methods:**

We selected and included 6,785 pediatric patients with a clinical diagnosis of bronchial asthma at the Children’s Hospital Affiliated to Shandong University from January 2018 to January 2025 with the cohort study design. Serum specific IgE (sIgE) was tested in all patients for 19 types of inhalant and food allergens.

**Results:**

In this study, house dust mites (HDMs) (52.99%) had the highest positive rate of, followed by molds (31.98%) and dog dander (28.17%). Food allergens that had the highest positive rates were egg white (32.45%), milk (19.96%) and beef (16.32%). The positive rate of the food allergens was higher in the 0–1 year-old group and the 1–3 year-old group compared to the inhaled allergens. On the other hand, inhaled allergens were more frequently positive than food allergens among children aged 3 to 6 years, 6 to 12 years, and over 12 years, with some of the differences being statistically significant. The positive rates of inhaled allergens were higher than those of food allergens in all months in terms of monthly distribution. Multi-allergen sensitization, in particular, the linking of HDMs with other allergens, was the most common sensitization profile identified in this study.

**Conclusion:**

The results of this study will be of great use in the transformation of experience-based diagnosis and treatment of childhood asthma into a standardized and personalized model on the basis of precise distribution of allergen. The change will reduce the occurrence of asthma attacks, enhance the quality of life of children and have much practical and clinical significance associated with the enhancement of the prevention and treatment of childhood asthma in the region.

## Introduction

One of the prevalent non-communicable diseases in children all over the world is bronchial asthma. It imposes an extensive health burden on the population and results in quality of life impairment in the children and the family members of the affected children (1, 2). Allergy sensitization is a powerful predisposing factor when it comes to asthma development and exacerbation in children, with a wide range of environmental allergens commonly eliciting and aggravating the asthma symptoms (3). Profiles of allergen sensitization vary widely by region or population under study (4). The differences are due to various exposures, various lifestyles and various genetically predisposed conditions of the population. These underscore the epidemiological evidence needed to control asthma. Our research was carried out in compliance with this requirement.

Allergic reactions caused are mediated by IgE which is involved in the development of bronchial asthma (5). Recent researches has estimated that approximately 40–50% of childhood asthma exhibits allergic sensitization (6). This indicates the significance of allergen-sIgE in the pathogenesis and progression of asthma. SIgE testing is essential in the diagnosis of allergy because it can be usefully used to objectively identify allergic sensitisation to multiple allergens (7).

The gap in knowledge regarding asthma pathophysiology is still substantial, despite the fact that significant progress has been made in studying the subject (8). Specifically, the regional differences in the rates of allergen sensitization in Chinese children (9). Although this type of data is abundant in western cohorts, which are well-characterized-inhalant triggers, such data is nonexistent in Chinese children. In addition, the research is limited in its scope and sample size (10). The age-specific change in the dominance of food to inhalant allergens might also use more characterisation. This has been associated with maturation of the immune. Sufficient profiling of sIgE, therefore, needs to be done in individual pediatric asthmatics. This is because this novelty of our study is highlighted by its huge sample and wide battery of 19 allergens likely to encompass both inhalants and foods (11), and the fact that our large investigation was carried out in a well-defined pediatric cohort in Shandong Province itself. With the technique, comprehensive and current characterization of the sIgE sensitization area with excellent contribution in regional level precision medicine is possible.

## Materials and methods

### Patients

A retrospective analysis was performed by collecting the demographic data and sIgE test results of 6,785 pediatric cases who were diagnosed with bronchial asthma at the Children’s Hospital affiliated to Shandong University during January 2018 to January 2025. The study was approved by the Ethics Committee of Children’s Hospital affiliated to Shandong University.

### Diagnostic criteria

According to the 2016 guidelines for prevention and treatment of bronchial asthma (12), the diagnostic criteria were as follows: (1) Recurrent wheezing, coughing, shortness of breath, and chest tightness are mostly associated with exposure to allergens, cold air, physical/chemical stimuli, respiratory infections, exercise, and hyperventilation (such as laughing and crying), often occurring or worsening at night and/or early morning; (2) During an attack, scattered or diffuse wheezing sounds dominated by the expiratory phase can be heard in both lungs, with prolonged expiratory phase; (3) The above symptoms and signs respond effectively to anti-asthma treatment or resolve spontaneously; (4) Other diseases causing wheezing, coughing, shortness of breath, and chest tightness should be excluded; (5) For individuals with atypical clinical manifestations (e.g., without obvious wheezing or wheezing sounds), at least one of the following criteria must be met: (1) Confirmation of reversible airflow limitation: Positive bronchodilator test: Forced expiratory volume in 1 s (FEV₁) increases by ≥12% 15 min after inhaling a rapid-acting *β*₂ receptor agonist (e.g., salbutamol metered-dose inhaler 200–400 μg); Improvement in pulmonary ventilation function after anti-inflammatory treatment: FEV₁ increases by ≥12% after 4–8 weeks of inhaled glucocorticoid and/or leukotriene antagonist therapy;(2) Positive bronchial provocation test;(3)Diurnal variation rate of peak expiratory flow (PEF) (continuous monitoring for 2 weeks) ≥ 13%. Asthma can be diagnosed in individuals who meet criteria 1–4 or criteria 4 and 5.

### Measurement of serum sIgE

The serum sIgE testing adopted the EUROLINE Atopy (China) (IgE) kit. Result interpretation: sIgE<0.35KUA/L is considered negative for allergens;sIgE≥0.35 KUA/L is considered positive for allergens. Since sIgE positivity for food allergens does not equate to clinical food allergy, we chose to use the objective criterion of sIgE ≥0.35 as the primary indicator for food allergen analysis. This decision was made to ensure data consistency and avoid subjective biases.

### Statistical analysis

We performed statistical analysis using SPSS (version 28.0) software. Heat map showing positive rates was created by OriginPro (Version: 2024) whereas bar chart and pie chart regarding positive rates were created by GraphPad Prism (Version: 9.5). The age categorical variables were classified into the groups of 0–1 year, 1–3 years, 3–6 years, 6–12 years and >12 years. Given that the variables analyzed are binary categorical data (positive/negative), the chi-square test was employed to compare the allergen positive rates across different age groups and months. Owing to the high number of comparisons (nineteen allergens, five age groups, and twelve months), Bonferroni correction applied for multiple comparisons. The Bonferroni correction is a statistical method employed to control Type I errors (false positives) in multiple comparisons. Its core logic lies in mitigating “spuriously significant” results arising from repeated comparisons by imposing a more stringent significance level threshold. The specific procedure is as follows: first, define the original significance level (*α*, typically set at 0.05) and the number of independent comparisons (n, i.e., the total count of independent inter-group comparisons conducted in the study); then compute the corrected significance level (*α*’) using the formula *α*’ = α / n. A difference is deemed statistically significant if the original *p*-value is less than α’; conversely, it is not statistically significant if the original *p*-value is greater than or equal to α’.

## Results

### Patients’ characteristics

A total of 6,785 children with a diagnosis of bronchial asthma were enrolled in the present study. Among the total number of students, there are more boys, which comprises of 66.68%. As for age, 74.45% of the subjects were between 3 and 12 years old. Seasonal evaluation indicated that 59.56% of occurrences registered in the second and third quadrants. In case of allergy positivity, the inhalation allergens showed a consistently higher positive rate than food allergens. Among the inhalant allergens, HDMs showed the highest positivity rate of 52.99 percent, followed by molds at 31.98%t and with dog dander at 28.17%. Egg white emerged as the most common allergenic food at 32.45%, followed by milk (19.96%) and beef (16.32%) (Table 1).

**Table 1:** The baseline of 6,785 children with asthma enrolled in this study.

Characteristic	***N***	%
Gender		
Female	2,261	33.32
Male	4,524	66.68
Ages (yrs)		
0–1	75	1.11
1–3	778	11.47
3–6	2,960	43.62
6–12	2092	30.83
>12	880	12.97
Month (Quarters)		
Q1	1,223	18.03
Q2	1935	28.52
Q3	2,106	31.04
Q4	1,521	22.41
Types of positive allergens		
Inhalant allergens		
Willow/Poplar/Elm	1,221	17.99
Common ragweed	1,375	20.27
Mugwort	1809	26.66
House dust mite	3,596	52.99
House dust	1,148	16.92
Cat dander	1,191	17.55
Dog dander	1911	28.17
Cockroach	1,119	16.49
Mold	2,170	31.98
Japanese hop	1,549	22.83
Food allergens		
Egg white	2,202	32.45
Milk	1,354	19.96
Peanut	914	13.47
Soybean	1,050	15.47
Beef	1,107	16.32
Mutton	884	13.03
Cod/Lobster/Scallop	975	14.37
Shrimp	725	10.69
Crab	880	12.97

### Distribution characteristics of inhalant and food allergens

**Figure 1:**
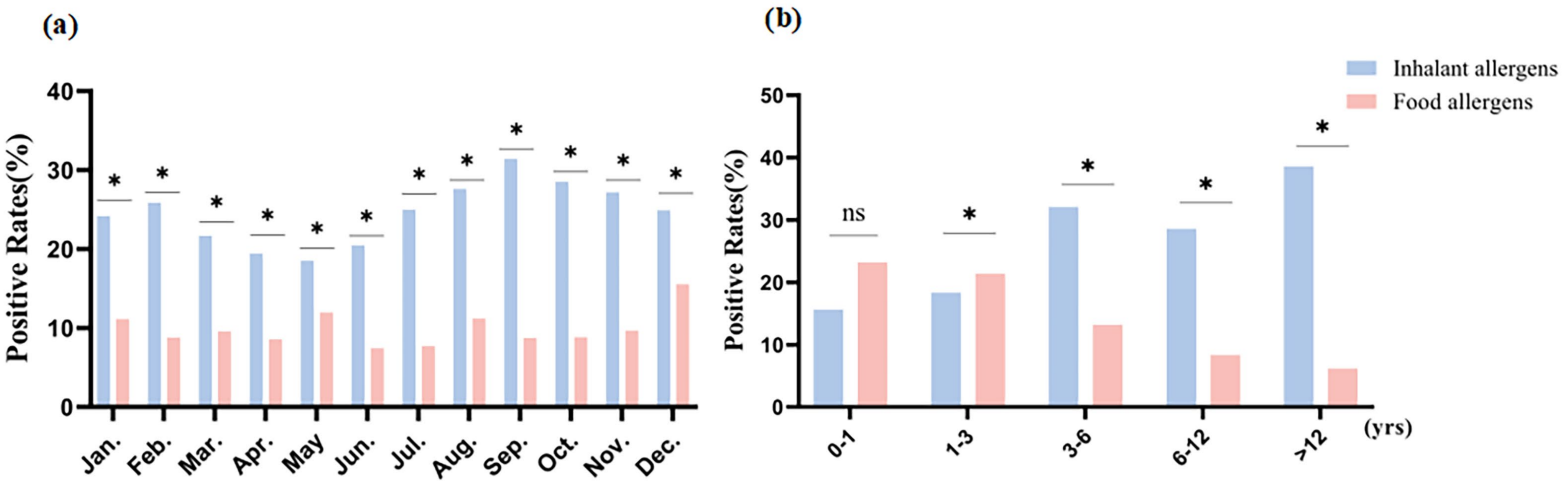
Distribution characteristics of inhalant and food allergens. * Original p-value < adjusted significance level (α′); ns: no statistical significance. **(a)** Distribution characteristics of inhalant and food allergens in different months; **(b)** Distribution characteristics of inhalant and food allergens in different age groups.

Bar charts to represent the distribution characteristics of inhalant and food allergens across different months and age groups (Figures 1a,b). In Figure 1a the total number of independent comparisons for the positive rates between inhalant and food allergens across different months is 12 following Bonferroni correction, the adjusted *α*’ = 0.05/12 ≈ 0.0042, the positive rates of inhaled allergens were consistently higher than those of food allergens across all months (original *p* < 0.001), with statistically significant differences observed in each month (original *p* < *α*’). In Figure 1b, the total number of independent comparisons for the positive rates between inhalant and food allergens across different age groups is 5, following Bonferroni correction, the adjusted *α*’ = 0.05/5 = 0.01, the positive rates of food allergens were higher than those of inhaled allergens in the 0–1 year-old group (original *p* = 0.082 > α’,there is no statistical significance in the difference) and 1–3 year-old group (original *p* = 0.002 < α’,the difference is statistically significant). Conversely, inhaled allergens exhibited higher positive rates than food allergens in the 3–6 year-old (original *p* < 0.001), 6–12 year-old (original *p* < 0.001), and >12 year-old groups (original *p* < 0.001), with statistically significant differences confirmed in all three groups (original *p* < *α*’).

### The heat maps of positive rates for inhalant and food allergens

**Figure 2:**
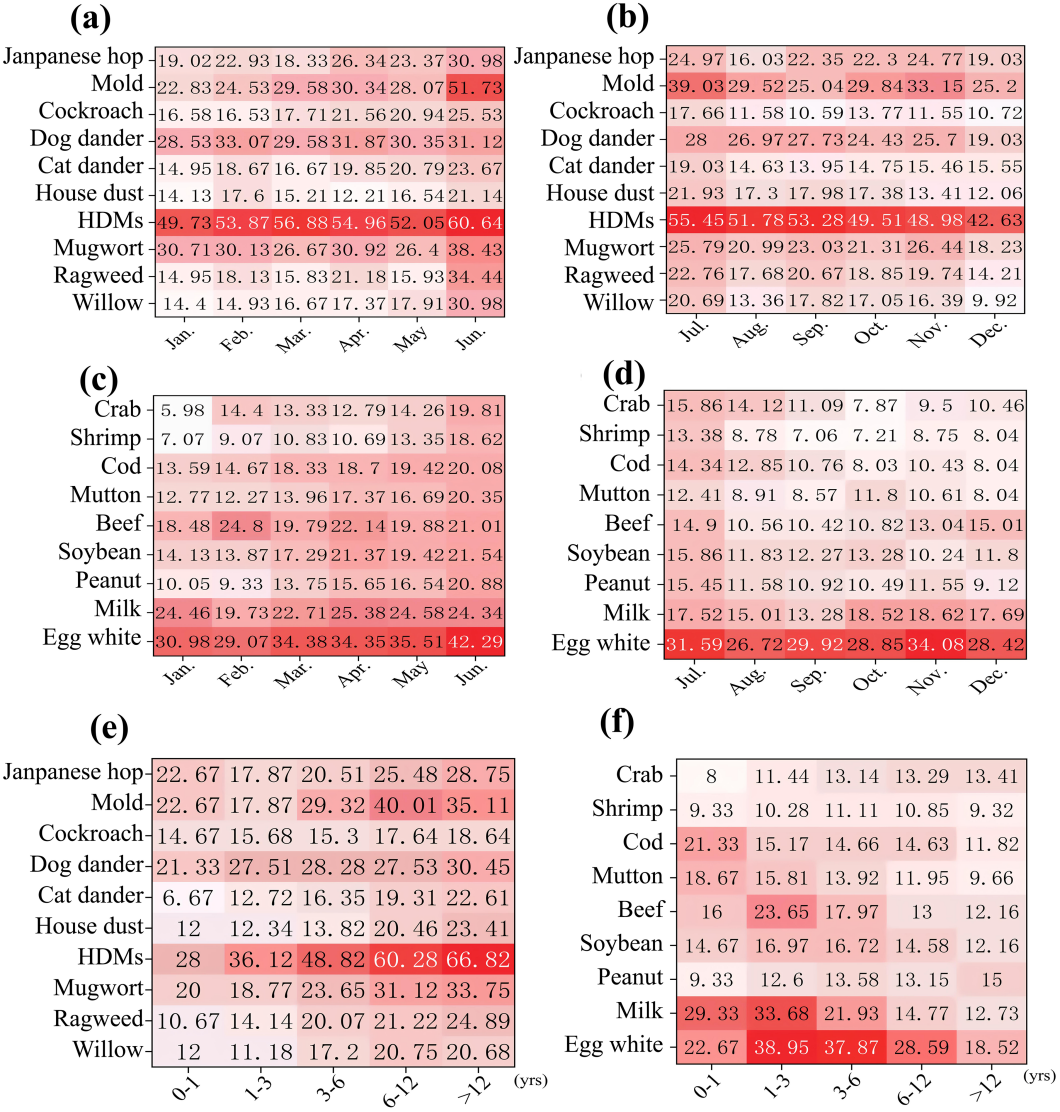
The heat maps of positive rates for inhalant and food allergens. **(a)** Positive rates of inhalant allergens distributed from January to June; **(b)** Positive rates of inhalant allergens distributed from July to December; **(c)** Positive rates of food allergens distributed from January to June; **(d)** Positive rates of food allergens distributed from July to December; **(e)** Positive rates of inhalant allergens distributed in different age groups; **(f)** Positive rates of food allergens distributed in different age groups.

We generated heat maps showing the positive rates of inhalants and food allergens during different months and age groups (Figures 2a–f). As the red of the boxes gets deeper, the value of the positive rate gets higher. Among food allergens, the positive rate of egg white was the highest while that of milk had the second-highest (Figures 2c,d,f). Specifically, in Figure 2f, the positive rate of egg white was highest (38.95%) in the 1–3 age group, followed by 37.87% in the 3–6 age group. The positive rate of milk was highest (33.68%) in the 1–3 age group, followed by 29.33% in the 0–1 age group. In Figures 2c,d, the positive rate of food allergens shows a peak in June, and the positive rate of food allergens in the first half of the year is higher than that in the second half of the year. For inhalant allergens, HDMs had the highest positive rate, followed by mold (Figures 2a,b,e). In Figure 2e, the positive rate of HDMs were highest (66.82%) in the >12 years age group, followed by 60.28% in the 6–12 years age group. The positive rate of mold was highest (40.01%) in the 6–12 years age group, followed by 35.11% in the >12 years age group. In Figures 2a,b, the positive rate of inhalant allergens showed a marked peak in June, while remaining relatively stable across other months of the year.

### Distribution of allergens sensitization patterns

**Figure 3:**
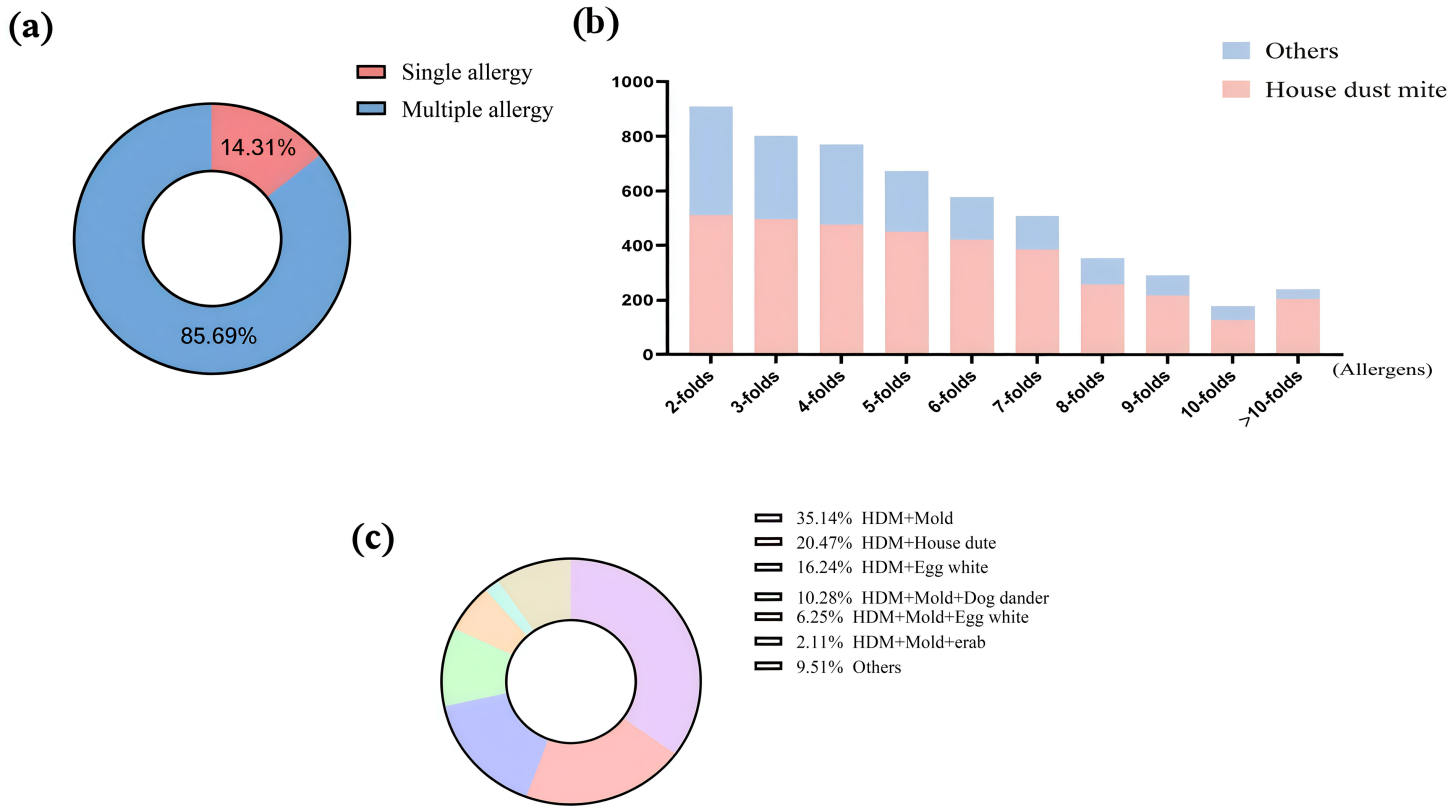
Distribution of allergens sensitization patterns. **(a)** Distribution of single sensitization and multiple sensitization; **(b)** Distribution of house dust mites in multiple sensitization; **(c)** Distribution of multiple sensitization patterns.

The sensitization pattern of allergens was predominantly characterized by multiple sensitization, accounting for 85.69% (Figure 3a). In cases of being sensitized to multiple allergens, sensitization to HDMs along with other allergens was very common (Figure 3b). For the HDMs-based polysensitization, the pattern of co-sensitization of HDMs along with mold was the primary pattern (35.14%); the pattern of co-sensitization of HDMs along with house dust was the secondary pattern (20.47%). Notably, the HDMs-mold combination was also the dominant cluster within the triple-allergen sensitization profiles (Figure 3c). In multivariate logistic regression analysis including multiple sensitization patterns with and without sensitization to HDMs, it was found that different age groups and preterm birth may be independent risk factors for these two sensitization patterns (Table 2).

**Table 2:** Multivariate logistic regression analysis of multiple sensitization patterns with and without HDMs sensitization.

Variables	β	S.E	Z	P	OR (95%CI)
Gender					
Female					1.00 (Reference)
Male	0.06	0.07	0.84	0.401	1.06 (0.92 ~ 1.22)
Age					
>12					1.00 (Reference)
0–1	−2.49	0.45	−5.54	<0.001	0.08 (0.03 ~ 0.20)
1–3	−1.39	0.19	−7.15	<0.001	0.25 (0.17 ~ 0.36)
3–6	−0.92	0.18	−5.18	<0.001	0.40 (0.28 ~ 0.56)
6–12	−0.44	0.18	−2.48	0.013	0.64 (0.45 ~ 0.91)
Month of asthma onset	−0.27	0.14	−1.93	0.054	0.76 (0.58 ~ 1.00)
Preterm birth status					
No					1.00 (Reference)
Yes	0.45	0.15	2.97	0.003	1.57 (1.17 ~ 2.12)

OR: Odds Ratio, CI: Confidence Interval.

### Analysis of differences in allergen sensitization in asthma complicated with allergic rhinitis

Allergic rhinitis is a common comorbidity of asthma. This study examined the disparity in the sensitization of allergens in asthma patients alone and asthma patients with allergic rhinitis. Table 3 indicates that the rate of polysensitization in asthma-with-allergic-rhinitis group (83.44%) was greater compared to that of asthma-alone group (74.38%). With regard to monosensitization, the sensitization rate of HDMs in asthma with allergic rhinitis group (58.44%) was more than that of the asthma-alone group (44.14%). In addition, out of the instances of polysensitization, the number of patients cross-sensitized to HDMs and mold was greater in the asthma-with-allergic-rhinitis group compared to the asthma-alone group. Each of the above differences was significant.

**Table 3:** Analysis of differences in allergen sensitization between the asthma-alone group and the asthma-complicated-with-allergic-rhinitis group.

Variables	Asthma-alone (*n* = 3,599)	Complicated with allergic rhinitis (*n* = 465)	Statistic	*P*
Sensitization type, *n*(%)			χ^2^ = 18.23	<0.001
Multiple allergy	2,677 (74.38)	388 (83.44)		
Single allergy	922 (25.62)	77 (16.56)		
Single allergy, *n*(%)			χ^2^ = 6.24	0.044
Others	290 (31.45)	16 (20.78)		
House dust mite	407 (44.14)	45 (58.44)		
Mold	225 (24.40)	16 (20.78)		
Multiple allergy, *n*(%)			χ^2^ = 12.09	<0.001
Others	569 (21.26)	53 (13.66)		
HDMs-mold combination sensitization	2,108 (78.74)	335 (86.34)		

χ^2^: Chi-square test.

## Discussion

According to our study, the asthmatic children of Shandong Province have a distinct allergen sensitization, a certain combination of inhalant and food allergens, which is quite dissimilar to other places in China and even some areas of Asia. When it comes to inhalant allergens, the main sensitizing agents are HDMs (52.99%), molds (31.98%), and dog dander (28.17%), and the sensitization to pollen and cockroaches is much lower than in cities like Shanghai and Tianjin (13, 14). In food allergens, egg white (32.45%), milk (19.96%) and beef (16.32%) are the predominant sensitizing agents in that order an arrangement that is starkly different than the seafood-dominant sensitization of Shanghai (seafood sensitization rate: 10.1%) and the diet-related pattern of sensitization in South Korea (milk sensitization rate: 30.2%) and Japan (seafood sensitization rate: 18.5%) (15). These distinctions are mainly ascribed to the distinct local climatic circumstances and the feeding arrangement. In terms of the findings above, this research has a crucial implication in advocating the change of models of childhood asthma diagnosis and treatment models the movement of empirical judgment to a standardized, individualized model based on the accurate data in the distribution of allergens.

Inhalant allergen profile among asthmatic children in Shandong Province is vastly different from that in Western countries and other Asian areas. According to our research, the main agents of sensitization are HDMs, molds, and dog dander. Conversely, the pollen and pet dander are among the most frequent inhalant allergens in Western cohorts (16, 17). This means that there are regional differences in allergen profiles, which closely correlate with the warm-temperate monsoon climatic conditions in Shandong, high temperature, and high humidity in summer (relative humidity 70–80%, temperature 25 °C–28 °C), which are highly conducive to the growth of HDMs and molds. In comparison, the western areas are drier with prolonged pollen seasons and grass and tree pollens are the predominant allergens there. Such results indicate that allergen profiles are regionally specific, and local climatic factors require specific environmental interventions.

Regarding food allergies, the most common food allergies among children in Shandong Province is egg white and milk. In our study, children aged≥3 years represent the highest prevalence of food allergens (with egg white sensitization rate of 38.95% in 1–3-year-olds). This trend is identical to the immature mucosal immune regulation of children younger than 3 years old (18), and the age-related rise in sensitization to food allergies is consistent with the hypothesis of the “allergic march” that postnatal sensitization to food allergens might be a precursor to the development of sensitization to inhalant allergens. The management of food allergies differs distinctly from that of inhalant allergies. For instance, by avoiding sensitizing antigens in young children (e.g., egg white and milk), emergency department visits associated with allergic exacerbations were prevented by 55% (19). Notably, this strategy is not a one-size-fits-all recommendation for all children. It is not a general intervention but a specific intervention that is aimed at asthma patients with food-related exacerbations, especially the ones whose symptoms worsen when they either drink or eat milk or egg white. In children with asymptomatic food sensitization, dietary restriction is not needed; children should be instructed to improve scrutiny of inhalant allergen exposure, as food sensitization may have an immune priming impact.

The seasonal distribution of the sensitization to inhalant allergens has a clear seasonal trend with the highest rates during June, which is the period of greater humidity in summer and more active reproduction of HDMs and molds. Conversely, the proportion of allergens to food is more positive during the first half of the year, which is likely related to the change in dietary structure according to season. Subsequent mechanistic research has validated a notable immune interaction between HDMs and moulds: the interaction enhances Th2-type inflammatory responses by means of TLR4 pathway signaling and cross-epitope effects, which indicates that environment-induced interactions are a central pathway leading to the development and exacerbation of asthma (20–22). This discovery does not only explain a high polysensitization rate (85.69%) in the current cohort but also reveals that interactions between environment-induced allergens are a major path underlying the development and exacerbation of asthma. With this immune synergy established, the future management of asthma patients must begin to change its single-allergen approach to multi-agent interventions. Long-term exposure to molds decrease the effectiveness of HDMs-specific immunotherapy; thus, it is essential to achieve better environmental regulation of dust mites and indoor molds to achieve an effective management of asthma. Besides, as the comorbid association between asthma and allergic rhinitis indicates strong association between these two diseases in the presence of HDMs-mold polysensitization, specific environmental interventions should be prioritized in the case of this group in clinical practice-including indoor humidity (to prevent mould growth) and dust mite-proof bedding. Such steps can not merely relieve the symptoms of allergic rhinitis, but also promote the prevention of asthma attacks triggered by comorbid allergies.

Restructuring the management of the asthma should be based on the above findings to proceed to a complex management model rather than single-allergen intervention. This move will significantly improve clinical outcomes by creating a precision medicine framework based on regional allergen distribution data (23, 24). Meanwhile, allergen profiling into treatment regimens should be implemented to support risk-stratified care: in infants and young children (0–3 years old), food allergies should be prioritized, and strict dietary avoidance of highly prevalent allergens (e.g., egg white and milk) should be primary, with at least one environmental control strategy used to address HDMs; in children and adolescents (over 3 years), inhalant allergens should assume central importance, and allergen-specific immunotherapy (AIT) should be highly recommended for HDMs. Moreover, “preemptive management” is needed to manage the June peak of asthma exacerbations, and that is optimization of controller medications and intensifying environmental interventions by the end of the spring. Since polysensitization is common in children with asthma, sIgE testing should be performed on pediatric asthma patients; in patients with complicated sensitization, HDMs should receive priority attention. Ultimately, individualized and combined management strategies can lead to a shift in asthma care-empirical treatment to precision prevention and management (25, 26).

The data collection for our study spanned the years 2018 to 2025, a period that included the global COVID-19 pandemic. It is therefore relevant to consider how public health interventions during the pandemic—such as lockdowns, school closures, extended time spent indoors, and widespread use of face masks—may have influenced both allergen exposure patterns and the present findings. The elevated rates of sensitization to indoor allergens, including HDMs and molds, may reflect increased exposure resulting from prolonged time spent in confined environments. In contrast, the comparatively lower sensitization rates to pollen could be partly explained by reduced outdoor exposure, potentially mitigated by mask-wearing. Although our study was not designed to analyze temporal changes related to the pandemic, the overall sensitization profile observed here likely emerges from the interaction between regional factors and the distinctive, population-wide behavioral shifts during this period. We plan to conduct future studies incorporating well-defined temporal comparisons to elucidate the long-term impact of the pandemic on the epidemiological characteristics of allergic sensitization in children.

Additionally, our study has two main limitations. First, the local clinical practice of only using sIgE tests on children with identifiable signs of allergy (e.g., eczema, food allergy) and not on non-asthmatics was also used as a limitation to the selection of non-asthmatics in this area as controls - a limitation in line with local clinical practice in which non-asthmatics do not routinely receive such tests. This has complicated finding a representative non-asthmatic control group, with a direct effect on the interpretation of results on “sensitization prevalence”: e.g. the reported inhalant and food allergen sensitization prevalence across months and age groups is the prevalence and distribution of sensitization in the asthmatic cohort. They cannot be contrasted with the general population to test whether asthmatic patients are much more sensitized than normal individuals, or whether sensitization predisposes them to the development of asthma.” It is necessary to explain that a critical difference exists between “sensitization prevalence among asthmatic patients” and “sensitization-associated asthma risk.” Second, being a single-center retrospective study, it might not be applicable to other areas or groups with varying demographic characteristics. To overcome these shortcomings, future studies will be optimized in two aspects: first, to ensure the association between the sensitization and asthma onset, both a case–control design will be considered, which will include non-asthmatics to act as controls, and second, multi-center prospective studies will be carried out to better establish the existing sensitization patterns in more cohorts and improve the generalizability of the findings.

## Conclusion

Our study offers detailed data on the sensitization of children with asthma in China to allergens, where house dust mite and egg white are predominating allergens. This also indicates age-dependent changes in distribution and seasonal patterns. The outcomes will be used to develop region-specific programmes to prevent and manage the asthma disease to develop precision medicine to be more targeted and effective in treatment.

## Data Availability

The raw data supporting the conclusions of this article will be made available by the authors, without undue reservation.

## References

[1] Asher MI Rutter CE Bissell K Chiang CY el Sony A Ellwood E . Worldwide trends in the burden of asthma symptoms in school-aged children: global asthma network phase I cross-sectional study. Lancet. (2021) 398:1569–80. doi: 10.1016/S0140-6736(21)01450-1, PMID: 34755626 PMC8573635

[2] Bateman ED Hurd SS Barnes PJ Bousquet J Drazen JM FitzGerald M . Global strategy for asthma management and prevention: GINA executive summary. Eur Respir J. (2008) 31:143–78. doi: 10.1183/09031936.00138707, PMID: 18166595

[3] Gans MD Gavrilova T. Understanding the immunology of asthma: pathophysiology, biomarkers, and treatments for asthma endotypes. Paediatr Respir Rev. (2020) 36:118–27. doi: 10.1016/j.prrv.2019.08.002, PMID: 31678040

[4] Newson RB van Ree R Forsberg B Janson C Lötvall J Dahlén SE . Geographical variation in the prevalence of sensitization to common aeroallergens in adults: the GA(2) LEN survey. Allergy. (2014) 69:643–51. doi: 10.1111/all.12397, PMID: 24654915

[5] Galli SJ Tsai M. IgE and mast cells in allergic disease. Nat Med. (2012) 18:693–704. doi: 10.1038/nm.2755, PMID: 22561833 PMC3597223

[6] Dharmage SC Perret JL Custovic A. Epidemiology of asthma in children and adults. Front Pediatr. (2019) 7:246. doi: 10.3389/fped.2019.00246 31275909 PMC6591438

[7] Matricardi PM Dramburg S Potapova E Skevaki C Renz H. Molecular diagnosis for allergen immunotherapy. J Allergy Clin Immunol. (2019) 143:831–43. doi: 10.1016/j.jaci.2018.12.1021, PMID: 30850070

[8] Papadopoulos NG Bacharier LB Jackson DJ Deschildre A Phipatanakul W Szefler SJ . Type 2 inflammation and asthma in children: a narrative review. J Allergy Clin Immunol Pract. (2024) 12:2310–24. doi: 10.1016/j.jaip.2024.06.010, PMID: 38878861

[9] Huang K Yang T Xu J Yang L Zhao J Zhang X . Prevalence, risk factors, and management of asthma in China: a national cross-sectional study. Lancet. (2019) 394:407–18. doi: 10.1016/S0140-6736(19)31147-X, PMID: 31230828

[10] D'Amato G Chong-Neto HJ Monge Ortega OP Vitale C Ansotegui I Rosario N . The effects of climate change on respiratory allergy and asthma induced by pollen and mold allergens. Allergy. (2020) 75:2219–28. doi: 10.1111/all.14476, PMID: 32589303

[11] Locke A Hung L Upton J O'Mahony L Hoang J Eiwegger T. An update on recent developments and highlights in food allergy. Allergy. (2023) 78:2344–60. doi: 10.1111/all.15749, PMID: 37087637

[12] The Respiratory Group of the Pediatric Society of Chinese Medical Association. Guidelines for the diagnosis and management of bronchial asthma in children (2016 edition). Chin J Pediatr. (2016) 54:167–81. doi: 10.3760/cma.j.issn.0578-1310.2016.03.003

[13] Huang C Liu W Hu Y Zou Z Zhao Z Shen L . Updated prevalences of asthma, allergy, and airway symptoms, and a systematic review of trends over time for childhood asthma in Shanghai, China. PLoS One. (2015) 10:e0121577. doi: 10.1371/journal.pone.0121577, PMID: 25875829 PMC4395352

[14] Luo S Sun Y Hou J Kong X Wang P Zhang Q . Pet keeping in childhood and asthma and allergy among children in Tianjin area, China. PLoS One. (2018) 13:e0197274. doi: 10.1371/journal.pone.0197274, PMID: 29768461 PMC5955563

[15] Leung TF Wong GW. The Asian side of asthma and allergy. Curr Opin Allergy Clin Immunol. (2008) 8:384–90. doi: 10.1097/ACI.0b013e3283103a8e, PMID: 18769189

[16] Idrose NS Dharmage SC Lowe AJ Lambert KA Lodge CJ Abramson MJ . A systematic review of the role of grass pollen and fungi in thunderstorm asthma. Environ Res. (2020) 181:108911. doi: 10.1016/j.envres.2019.108911, PMID: 31759647

[17] Lappe BL Ebelt S D'Souza RR Manangan A Brown C Saha S . Pollen and asthma morbidity in Atlanta: a 26-year time-series study. Environ Int. (2023) 177:107998. doi: 10.1016/j.envint.2023.107998, PMID: 37290290 PMC10600739

[18] Lloyd CM Saglani S. Development of allergic immunity in early life. Immunol Rev. (2017) 278:101–15. doi: 10.1111/imr.12562, PMID: 28658545

[19] Durban R Groetch M Meyer R Coleman Collins S Elverson W Friebert A . Dietary Management of Food Allergy. Immunol Allergy Clin N Am. (2021) 41:233–70. doi: 10.1016/j.iac.2021.01.009, PMID: 33863482

[20] Meloun A León B. Sensing of protease activity as a triggering mechanism of Th2 cell immunity and allergic disease. Front Allergy. (2023) 4:1265049. doi: 10.3389/falgy.2023.1265049, PMID: 37810200 PMC10552645

[21] Yang J Zhang M Luo Y Xu F Gao F Sun Y . Protopine ameliorates OVA-induced asthma through modulatingTLR4/MyD88/NF-κB pathway and NLRP3 inflammasome-mediated pyroptosis. Phytomedicine. (2024) 126:155410. doi: 10.1016/j.phymed.2024.155410, PMID: 38367422

[22] Howie D Mauro C. Editorial: metabolism and immune tolerance. Front Immunol. (2018) 9:2678. doi: 10.3389/fimmu.2018.02678, PMID: 30498502 PMC6249368

[23] Phillips EJ Walter JE. Precision medicine in allergy and immunology through the Lens of Immunogenomics. J Allergy Clin Immunol Pract. (2022) 10:1776–7. doi: 10.1016/j.jaip.2022.05.025, PMID: 35809990

[24] Boyd SD Hoh RA Nadeau KC Galli SJ. Immune monitoring for precision medicine in allergy and asthma. Curr Opin Immunol. (2017) 48:82–91. doi: 10.1016/j.coi.2017.08.007, PMID: 28889067 PMC5743231

[25] Virchow JC. Allergen immunotherapy (AIT) in asthma. Semin Immunol. (2019) 46:101334. doi: 10.1016/j.smim.2019.101334, PMID: 31711771

[26] Batard T Taillé C Guilleminault L Bozek A Floch VB Pfaar O . Allergen immunotherapy for the prevention and treatment of asthma. Clin Exp Allergy. (2025) 55:111–41. doi: 10.1111/cea.1457 39363801 PMC11791393

